# Preventing enzymatic browning of freshly cut green bananas through immersion in normal water, lemon juice, and coconut water

**DOI:** 10.1002/fsn3.4284

**Published:** 2024-06-25

**Authors:** Shampa Sarkar, Sumaia Akhter, Joysree Roy, Md. Abdul Wazed, Raihan Abedin, Suvrow Neogie, Khairul Bashar Mishat, Md. Sazzat Hossain Sarker

**Affiliations:** ^1^ Department of Food Processing and Preservation Hajee Mohammad Danesh Science and Technology University Dinajpur Bangladesh; ^2^ Department of Food Engineering and Technology Hajee Mohammad Danesh Science and Technology University Dinajpur Bangladesh

**Keywords:** enzymatic browning, fresh‐cut green banana, natural anti‐browning agents, PPO activity

## Abstract

The rising demand for freshly cut agricultural produce like bananas, apples, pears, potatoes, and roots due to health concerns and modern lifestyles has heightened awareness of their susceptibility to browning, which diminishes their appeal and contributes to food waste. The present study explored the efficacy of natural anti‐browning agents in prolonging the quality and shelf‐life of freshly cut green banana slices. The bananas underwent treatment with normal water (NW), lemon juice (LJ), and coconut water (CW), and the changes in physicochemical properties, such as browning intensity, color, firmness, total soluble solid (TSS), total phenolic content (TPC), total flavonoid content (TFC), and also microbial attributes, were evaluated during their storage at 4 ± 1°C for 12 days. The results demonstrated significant enhancements in treated samples compared to untreated ones. While normal water and lemon juice‐treated banana slices exhibited visual browning during storage, coconut water immersion proved superior in maintaining visual appeal, whiteness, and lightness while delaying yellowing and browning. Coconut water‐treated samples also displayed firmer texture (0.75 kg), lower TSS (5.67 °Brix), and reduced weight loss (9.14%) after 12 days, in contrast to samples subjected to lemon juice and normal water treatments which showed lesser texture (0.68 kg, 0.58 kg), higher TSS (5.87 °Brix, 6.10 °Brix), and greater weight loss (11.76%, 16.09%), respectively. Furthermore, coconut water‐treated samples retained higher levels of total phenols (392.67 mg GAE (gallic acid equivalent)/100 g FW (fresh weight), total flavonoids (55.67 mg QE (quercetin equivalent)/100 g FW), and 2,2‐diphenyl‐1‐picrylhydrazyl (DPPH) scavenging activity (38.68%). Coconut water treatment also significantly suppressed polyphenol oxidase (PPO) activity (14 U/g) compared to lemon juice (16 U/g) and normal water (26 U/g) treatments, and untreated samples (133 U/g) after 12 days. Additionally, microbial load remained within acceptable limits for all samples, with coconut water‐treated samples showing the lowest values. Thus, coconut water is a promising natural solution for inhibiting browning and preserving the quality of fresh‐cut green banana slices during storage.

## Graphical abstract

This study explores natural anti‐browning agents' efficacy in preserving fresh‐cut green bananas. Treatments with normal water, lemon juice, and coconut water were compared over a 12‐day storage period at 4 ± 1°C. Results indicate that coconut water immersion significantly improves visual appearance, firmness, and biochemical quality, effectively reducing browning and microbial load compared to other treatments and raw samples. Coconut water emerges as a promising natural solution for extending the shelf‐life and quality of fresh‐cut produce.

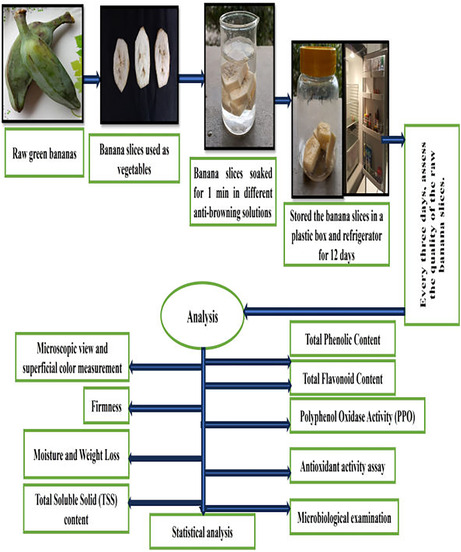

## 1 INTRODUCTION

Fresh‐cut food refers to any raw fruit or vegetable that has been physically changed after harvest without further processing such as cooking or blanching (FDA, 2018). The sector of horticulture that deals with minimally processed or fresh‐cut fruits and vegetables is known to be a robust and prosperous part of the business (Ali et al., 2023), fueled by increasing customer demand for their superior nutritional value or pure nutritional source, convenience, and fresh appearance (Yousuf et al., 2018). Among all fresh‐cut vegetables, banana (*Musaceae*) is a very demandable climacteric fruit that holds significant economic value in both local and international markets across the globe throughout the whole year.

The green banana is widely recognized as a valuable dietary source of various essential elements, such as bioactive phenols, antioxidants, and potassium (Youryon & Supapvanich, 2017). Moreover, green bananas can produce a variety of food products like cheese bread, juice, pasta, etc. (Marques et al., 2017; Zandonadi et al., 2012). In Bangladesh, the production of bananas in 2021–2022 was 826,179.62 metric tons (BBS, 2022). Besides this huge production, the most significant barrier to marketing fresh‐cut fruits and vegetables including green bananas is their relatively limited shelf‐life due to tissue softening and browning. The postharvest losses of bananas during storage, marketing, and transportation can range from 25% to 50% in the whole world (Al‐Dairi et al., 2023). In addition, from harvest to consumption, the gross postharvest losses of bananas in Bangladesh were 21.67% of the total yield (Saha et al., 2021).

Unit operations like grading, washing, sorting, peeling, slicing, chopping, and packing are used to develop fresh‐cut or minimally processed fruits and vegetables. These operations lead to quality deterioration, which is exhibited by cut‐surface browning, microbial contamination, moisture loss, increased respiration, softening, and ethylene evolution, and also decreased nutritional value as compared to whole fruit (Graca et al., 2015). The quality, especially the color of freshly cut produce, like green bananas, is a significant feature that affects its excellence. Banana slices that have just been cut tend to discolor and soften more quickly than uncut ones due to enzymatic browning (Vilas‐Boas & Kader, 2006) and 50% of the fresh fruit waste due to color degradation (Arnold & Gramza‐Michałowska, 2022). The fresh produce industry thus endures enormous financial losses and controlling browning can decrease economic losses and food waste.

In enzymatic browning reaction, oxidative reactions occur due to the phenolic compounds induced by polyphenol oxidase (PPO) activity underneath aerobic conditions, which is mostly liable for the browning of fresh‐cut fruits and vegetables (Ayaz et al., 2008; Wessels et al., 2014) and affects the product's commercial efficiency. The injuries or cuts to the tissues permit the internal release of nutrients and enzymes that enhance enzymatic activity and the development of organisms. In addition, enzymatic browning is one of the major issues that constrain the period of storage of fresh goods. Hence, protecting fruits and vegetables from oxidation during processing and storage is the uppermost priority for the food industry. There are various approaches for controlling enzymatic browning in vegetables and fruits, including the heating and cooling method, heat shock method, high‐pressure processing, osmotic dehydration, adding chemical anti‐browning agents, using edible coatings, and putting them in packages with a modified atmosphere (Alam et al., 2020; Anyasi et al., 2015; Ayaz et al., 2008; Bico et al., 2009; Singh et al., 2018; Yildiz, 2018). The most auspicious strategy to stop the browning and softening of fruits and vegetables is to utilize chemical anti‐browning agents like chelating agents, acidulants, cyclodextrins, and antioxidants due to their low‐cost and high‐reducing properties (Vilas‐Boas & Kader, 2006). Ascorbic acid and its derivatives are frequently employed for preventing browning on fresh‐cut fruits and vegetables, yet these effects are transitory. Also, sulfur dioxide and sulfites can be used to achieve long‐term anti‐browning effects. Nevertheless, the process of sulfiting might hinder the absorption of thiamine by breaking down the vitamin, thereby causing asthmatic responses and cardiovascular symptoms in susceptible individuals (Rangan & Barceloux, 2009). The US Food and Drug Administration (FDA) restricted the use of sulfites on fruits and vegetables meant to be served raw or displayed fresh to the public (Timbo et al., 2004). Moreover, consumers want to eat chemical‐free foods due to their health concerns and alternative methods are being explored to increase the shelf‐life of fresh‐cut fruits. Therefore, the substitution of these compounds efficient in preventing browning constitutes a significant concern for the researchers and food industries, particularly natural compounds and their derivatives that are safe for human health and eco‐friendly.

The use of natural anti‐browning agents is so effective at preventing browning and improving the safety of food and the preservation of the environment that their application is increasing in prevalence. These natural compounds have secondary plant metabolites that have antioxidant properties, and they may be used as reducing agents (Wessels et al., 2014). Pineapple juice, rhubarb juice, coconut water, lemon juice, honey, normal water, and green tea extract have shown encouraging outcomes in preventing fresh‐cut fruit including banana discoloration on cut surfaces (Chaisakdanugull et al., 2007; Supapvanich et al., 2020; Wessels et al., 2014). A study observed that lemon juice possesses anti‐browning qualities because it has citric acid and ascorbic acid (Moon et al., 2020). According to the previous study, coconut water includes 3%–7% sugar, high potassium (K), ascorbic acid, estrogen‐like chemicals, and phenolic compounds like catechin and salicylic acid, which have antioxidant and reducing characteristics (Mahayothee et al., 2016). However, based on our knowledge from the previous study, no one has used normal water, lemon juice, or coconut water in a single study to prohibit enzymatic browning. Therefore, the current study was designed to prevent the enzymatic browning of mature fresh‐cut green bananas utilizing natural anti‐browning substances such as normal water (which was potable tap water), lemon juice, and coconut water and investigate the effect of these anti‐browning agents on the storage quality of green banana slices.

## 2 MATERIALS AND METHODS

### 2.1 Sample collection and preparation

Bananas with *Musa ornata* variety at Stage 1 (the peel was completely green and the fruit was hard) and free of visible damage were collected from Basherhat, Dinajpur, Bangladesh. Mature golden Malayan Dwarf coconut (*Cocos nucifera*) and Kagzi lemon (*Citrus aurantifolia*) were also purchased from the local market of Dinajpur. The green bananas were washed with normal water for cleaning and removal of extraneous material and were peeled manually with the knife and then cut into 2.5‐mm thick slices.

### 2.2 Treatment with natural anti‐browning agents

Due to the increasing desire to use natural resources to produce safer and healthier foods, in this study, normal water (pH: 7.36, TSS: 0.1 °Brix), lemon juice (pH: 2.64, TSS: 5.8 °Brix), and coconut water (pH: 4.50, TSS: 5.1 °Brix) were used as natural anti‐browning agents. The sliced bananas were immediately immersed in normal water (500 mL), lemon juice (500 mL), and coconut water (500 mL) along with raw bananas without dipping in any solution (control sample) for 1 min at room temperature (28 ± 2°C). Then the solutions were immediately drained, and instantly the samples were kept in a plastic container and stored at 4 ± 1°C for 12 days. Triplicates for every experiment of each treatment were carried out in every 3‐day intervals (0, 3rd, 6th, 9th, and 12th days) during the storage period for further experiments.

### 2.3 Quality assessment of freshly cut green banana slices

#### 2.3.1 Visual appearance, microscopic view, and superficial color measurement

Using a compound microscope (Microscope Series IM‐660; Spectralab Scientific Inc., Germany) and a camera, respectively, the microscopic and visual views of green banana slices were captured over the storage time. The Colorimeter (Minolta, CR‐300, Osaka, Japan) was utilized to measure the color of banana slices. Slices of bananas were tested for L*, a*, and b* values. The whiteness index (WI) (Equation 1) and browning values (Equation 2) were determined using the equations of Supapvanich et al. (2020), as shown below.

(1) $$\text{Whiteness index},\mathrm{WI}=100-\sqrt{\left\{{\left(100-L*\right)}^{2}+a{*}^{2}+b{*}^{2}\right\}}$$

(2) $$\text{Browning value},\mathrm{BV}=\frac{100\times \left(X-0.31\right)}{0.172}$$

where *X* = (a* + 1.75 L*)/(5.645 L* + a*‐3.012b*).

#### 2.3.2 Firmness

Banana slice firmness was assessed utilizing a penetrometer integrated into a test stand, employing a cylindrical probe with a diameter of 2 mm (manufactured by Wagner, USA). Firmness, defined as the maximum force (in kilograms) required until tissue failure (Soltani et al., 2010), was measured at the midpoint of the banana, situated between the stem end and the distal end.

#### 2.3.3 Moisture content and weight loss

The moisture content of banana slices was examined by a digital moisture analyzer (Model: XY‐105MW, China). After every 3 days, the weight of every banana slice was determined using an electronic balance (AS 220.R2, Radwag, Poland) with a precision of 0.001 g. The percentage of weight loss (WL) was calculated using the following equation (Kumar et al., 2018):

(3) $$\mathrm{WL}=\left[\frac{\left(\mathrm{IW}-\mathrm{EW}\right)}{\mathrm{IW}}\right]\times 100$$

where WL stands for weight loss (%), IW for the initial weight (g), and EW for the end weight (g).

#### 2.3.4 Total soluble solid

A digital refractometer (HI‐96801, Italy) was used to measure the total soluble solid (TSS) and was expressed as the °Brix.

#### 2.3.5 Total phenolic content

The method outlined by Supapvanich et al. (2020) was employed with slight modifications to determine the total phenolic content of banana slices' surface tissue. Initially, 2 g of fresh sample was homogenized for 3 min with 60% (v/v) ethanol, followed by filtration through Whatman No. 1 filter paper. Subsequently, 2 mL of saturated sodium carbonate (Na_2_CO_3_) solution was added to 1 mL of filtrate, along with 1 mL of 50% (v/v) Folin–Ciocalteu reagent. The mixture was vortexed and allowed to stand at room temperature for at least 30 min. Afterwards, the absorbance of the solution was measured at 750 nm using a spectrophotometer (UV‐1800; Shimadzu Corporation, Kyoto, Japan). The total phenol content was calculated using a linear equation derived from the gallic acid (GA) standard curve, and the results were reported in milligrams of gallic acid equivalent per gram of fresh weight (mg GAE/100 g FW).

#### 2.3.6 Total flavonoid content

The total flavonoid content (TFC) was assessed through a colorimetric method, as described by Kabir et al. (2021). TFC values were determined using a calibration curve with quercetin standards and expressed as milligrams of quercetin equivalents per 100 g of fresh weight (mg QE/100 g FW). In a 15 mL falcon tube, 1 mL of extract, 4 mL of distilled water, and 0.3 mL of 5% sodium nitrite (NaNO_2_) solution were combined and left for 5 min. Then, 0.3 mL of 10% aluminum chloride (AlCl_3_) was added and left for an additional minute. A total volume of 2 mL of distilled water was mixed with 2 mL of a 1 M sodium hydroxide (NaOH) solution, and the resulting solution was thoroughly mixed. After centrifugation at 4000 rpm (revolutions per minute) for 10 min, the tubes were incubated in the dark at room temperature for 15 min. TFC was calculated in milligrams of quercetin equivalents per 100 g of fresh weight (mg QE/100 g FW) by measuring the absorbance at 510 nm using an ultraviolet–visible (UV–vis) spectrophotometer (UV‐1800, Shimadzu Corporation, Kyoto, Japan), with a quercetin standard curve ranging from 0 to 100 μM.

#### 2.3.7 Antioxidant activity assay

The 2,2‐diphenyl‐1‐picrylhydrazyl (DPPH) scavenging power of banana slices was determined using the Youryon and Supapvanich (2017) technique. The reaction was initiated by mixing 0.5 mL of 1 mM DPPH in methanol with 5 mL of diluted supernatant. After recording the absorbance at 515 nm for 0 min, the mixture was left in the dark for 5 min. The following equation was utilized to determine the DPPH free radical's scavenging capacity:

(4) $$\text{DPPH free radical scavenging activity},\%=\left[\frac{\left(A_{0}-A_{10}\right)}{A_{0}}\times 100\right]$$

where sample absorbance at 0 min is denoted by *A*_0_ and sample absorbance at 10 min is indicated by *A*_10_.

#### 2.3.8 Polyphenol oxidase activity

The activity of polyphenol oxidase (PPO) was measured using the approach based on Galeazzi et al. (1981), with minor adjustments. A 5 g sample was obtained and thoroughly mixed in a mortar and pestle with 10 mL of a 0.2 M sodium phosphate buffer solution at a pH of 7.0. The sample was placed in a falcon tube and subjected to vortexing for 5 min. After that, the material was centrifuged for 10 min at 10,000 *g* at 4°C. The crude enzyme extract was made from the collected supernatant. Three milliliters in total was used for the reaction, which included 2 mL of 0.1 M catechol, 0.5 mL of distilled water, and 0.5 mL of sample extract in a cuvette. With a 30‐s interval, absorbance was measured at 420 nm using an ultraviolet–visible (UV–vis) spectrophotometer (UV‐1800; Shimadzu Corporation, Kyoto, Japan). The data were quantified as units per gram of fresh weight (U/g).

#### 2.3.9 Microbiological examination

Microbes were counted in raw and treated banana pieces using a method described by Fatima et al. (2016). In the laboratory, serial dilution was done by mixing 1 g of ground raw and treated sample with 9 mL of distilled water. Then, the method was serially done 10 times to dilute the material and ensure consistent findings. Twenty milliliters of agar media (nutrient agar) was then poured into the Petri dishes and left to solidify to prepare the culture media. Then a 0.1 mL diluted sample was transferred from each test tube to petri dishes, which were typically rotated to spread the microbial culture.

After covering the Petri dishes with parafilm and placing them in an inverted position, the sample was incubated (IN55, Memmert GmbH & Co. KG, Germany) at 37°for 24–48 h. It took 24 h to count the colonies in each petri dish and figure out the colony‐forming unit per milliliter (CFU/mL). Using the following equation, the total plate count was found as:

(5) $$\frac{\mathrm{CFU}}{\mathrm{mL}}=\frac{\text{Number of colonies}\times \text{Dilution factor}}{\text{Amount plated}}$$

### 2.4 Statistical analysis

These experimental data were analyzed using the statistical program SPSS (IBM/version 27). The factors involved in the study were banana slices treated with various immersions and the duration of storage. If a significant disparity in means was detected by the analysis of variance (ANOVA), subsequent post hoc comparisons were conducted using one‐way ANOVA to assess the means of the responses for the treatment and storage. Statistically significant differences were defined as *p* ≤ .05 among the samples. Pearson's correlation coefficient was performed by using Origin Pro2024 software to assess the correlations between the L* value, whiteness index, browning value, TPC, TFC, DPPH scavenging activities, and PPO activity.

## 3 RESULTS AND DISCUSSION

### 3.1 Visual and microscopic characteristics of banana slices

**FIGURE 1:**
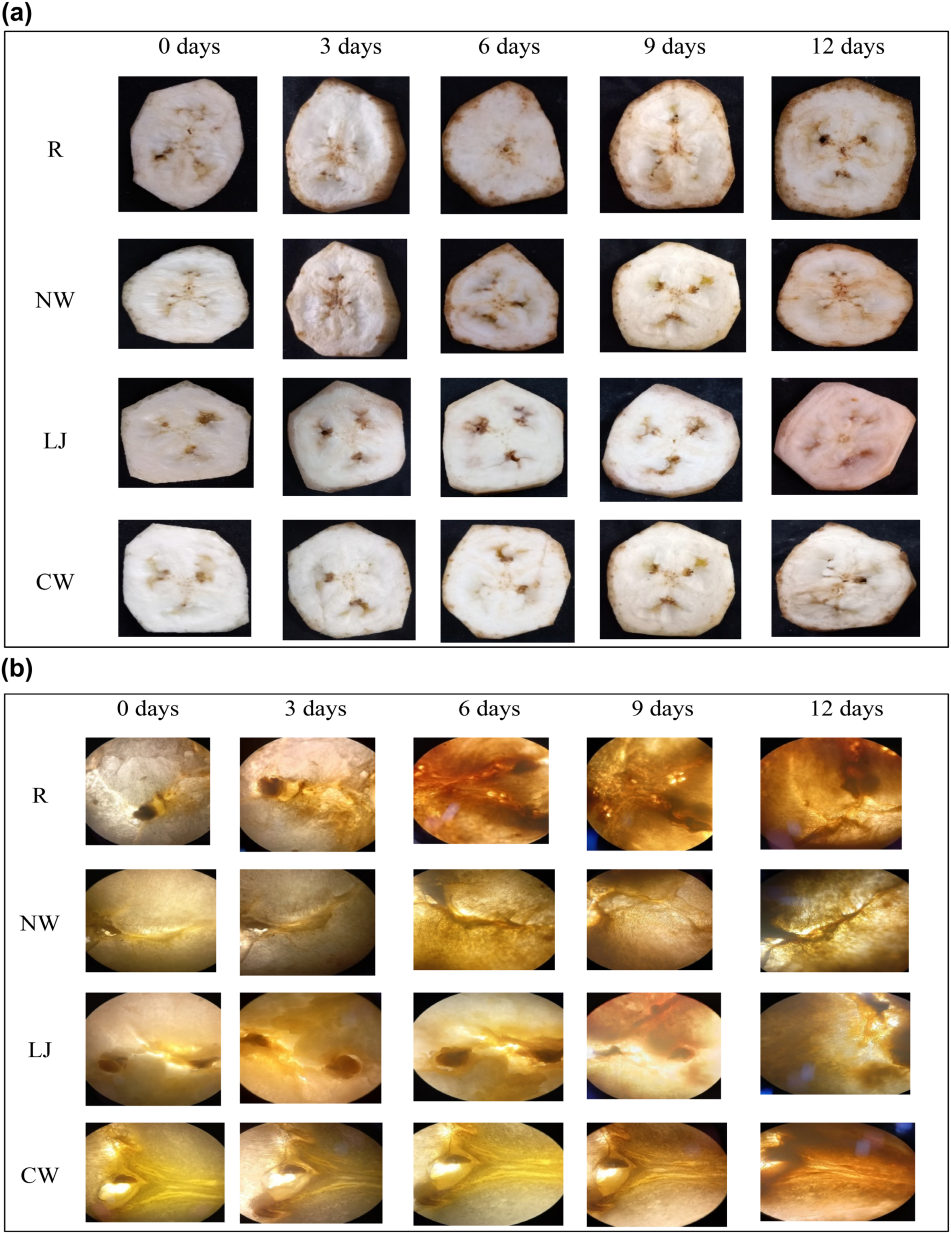
(a) Visual appearance of banana slices with different treatments in storage days. (b) Microscopic appearance of banana slices with different treatments in storage days. CW, coconut water; LJ, lemon juice; NW, normal water; R, raw.

Figure 1a,b depict the visual and macroscopic characteristics of raw and treated banana slices. In contrast to the raw and normal water immersions, the lemon juice and coconut water immersions prevented banana slices from browning during storage. There was a noticeable browning of the raw samples within a short period following the treatment (day 0) in Figure 1a; however, the banana slices immersed in normal water, lemon juice, and coconut water did not show any signs of browning. When compared to hypanthium, the browning occurring in the mesocarp was visible when viewed through a microscope, as shown in Figure 1b. It is commonly known that after slicing, the center of banana slices browns more quickly than the flesh. This implies that compared to hypanthium, the banana mesocarp is more susceptible to enzymatic browning reactions. Similar to this, the heart‐brown, also known as the internal browning of a banana, is a physiological condition that typically appears around the center. This might be due to PPO activity, which was also observed mostly in apple core tissue by Supapvanich et al. (2020). Both lemon juice and coconut water‐immersed banana slices showed a slight rise in browning during the storage period than raw and normal water immersions. However, the coconut water‐immersed banana slices' browning was noticeably lower than that of lemon juice‐immersed banana slices. This might be due to the presence of reducing properties in coconut water that prevent oxidative reactions including enzymatic browning. Similar findings were reported by Supapvanich et al. (2020), who discovered that apple wedges submerged in coconut water had a lower incidence of browning than control samples. This finding demonstrated the effectiveness of coconut water immersions in preserving the aesthetic appeal and preventing the browning of banana slices while they are in refrigerated storage.

### 3.2 Superficial characteristics of color

**FIGURE 2:**
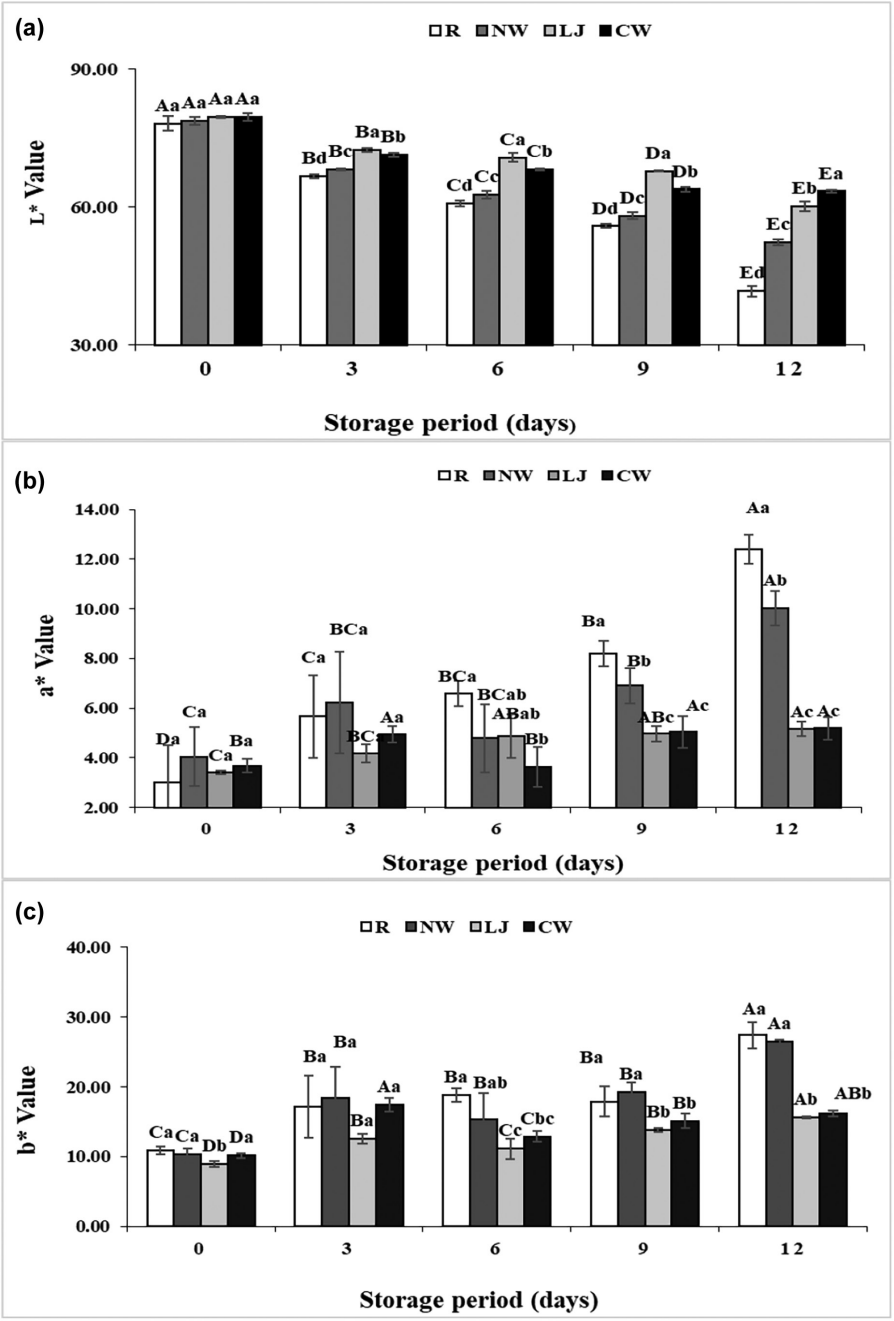
L* (a), a* (b), and b* (c) values of banana slices immersed in normal water (NW), lemon juice (LJ), and coconut water (CW) with the raw sample during storage at 4 ± 1°C for 12 days. Means followed by different superscript letters (a–d) are significantly different among treatments. Means followed by different superscript letters (A–E) are significantly different among storage time.

Figure 2 represents the effects of normal water, lemon juice, and coconut water immersions on the superficial color changes of banana slices compared to that of the raw sample. Except for the first‐day (0‐day) samples, every treated banana slice had an L* (lightness) value that was significantly (*p* ≤ .05) higher than that of the raw sample during the whole storage period (Figure 2a). The L* value of the banana slices was evidently maintained in both the lemon juice and coconut water immersions, and it was considerably greater than those of the raw and normal water‐immersed samples (*p* ≤ .05). This could be due to the fact that lemon juice and coconut water contain ascorbic acid, which has anti‐browning characteristics. The results of this study were consistent with a previous report by Yildiz (2018) on banana samples, in which the experiment was conducted with ascorbic acid and many other chemical treatments and found the highest L* value in ascorbic acid treatment. Furthermore, the L* value of banana slices immersed in coconut water was much higher than that of lemon juice immersion when comparing the two types of immersions. This could be because coconut water has a more effective antioxidant ingredient that can scavenge superoxide (Santos et al., 2013). In terms of a* value, as shown in Figure 2b, it is observed that a* value was increased in all samples during the storage period. It was also found that a* value was higher in normal water at zero days but in raw sample at 12 days. The lowest value of a* was found in lemon juice and coconut water immersions. However, a* value of banana slices in lemon juice and coconut water immersions was not significantly different on the 9th day and 12th day. Likewise, an increased b* value was also observed in all treatments throughout the storage (Figure 2c). The b* value was higher in raw samples and lower in coconut water‐immersed samples on the 12th day. Both lemon juice and coconut water immersions obviously delayed the increase in the b* value of banana slices compared to others. The significantly lower L* and higher a* and b* values of raw samples compared to those of lemon juice and coconut water‐treated banana slices indicated that during storage, there was an increase in the incidence of browning of raw banana slices. The results are in accordance with the results of Yildiz (2018) who found that L* values were lower and a* and b* values were higher in control during the storage period. Moreover, Supapvanich et al. (2020) observed higher L* and lower a* and b* values in coconut water‐treated apple wedges than in control samples during the storage period, which was consistent with the result of this study. Due to its high antioxidant content, coconut water is particularly effective in combating free radicals and superoxide radicals (Supapvanich et al., 2018).

### 3.3 Whiteness index and browning value

**FIGURE 3:**
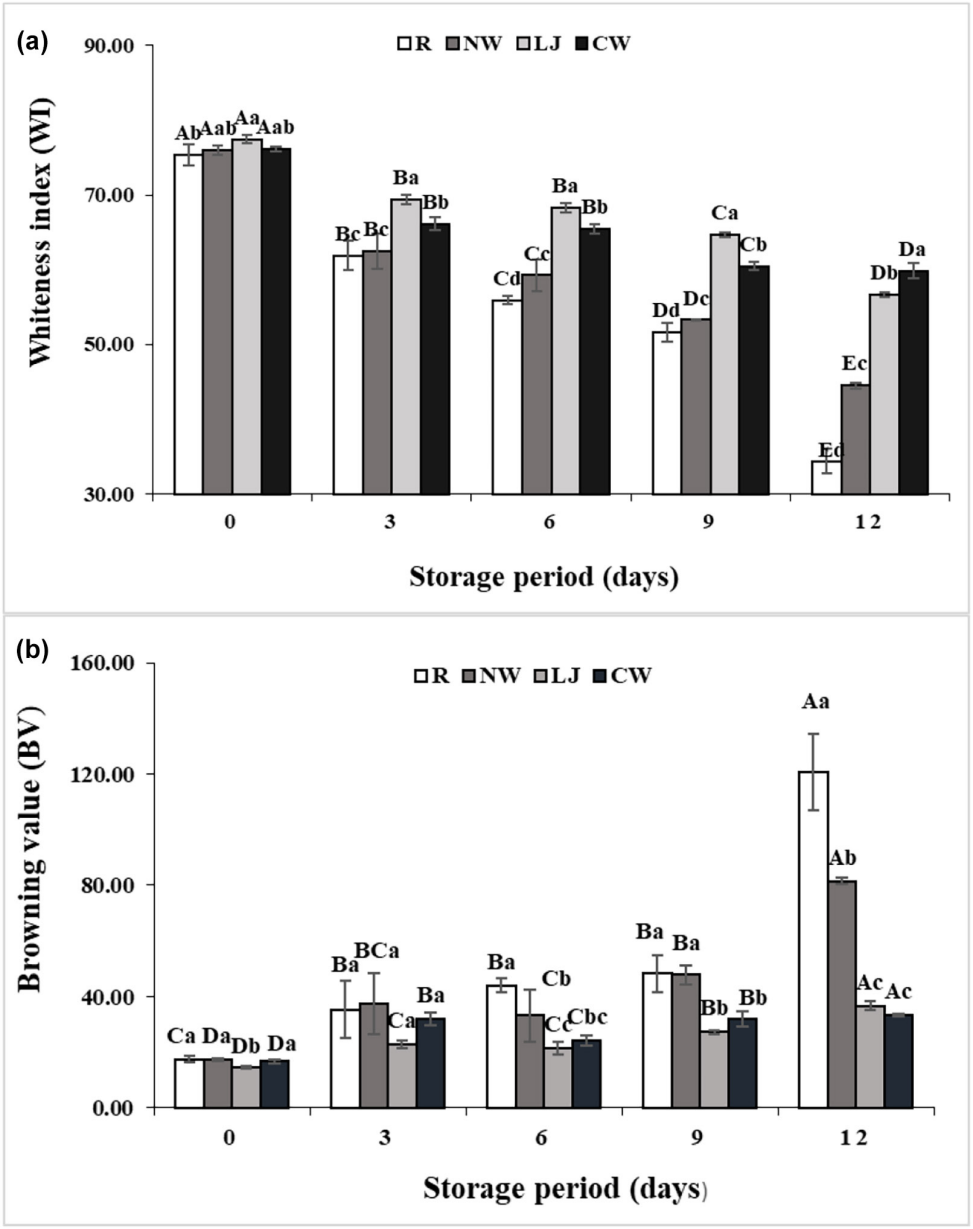
Whiteness Index (a) and Browning Value (b) of banana slices treated with normal water (NW), lemon juice (LJ), and coconut water (CW) with the raw sample during storage at 4 ± 1°C for 12 days. Means followed by various superscript letters (a–d) show significant differences among treatments. Means followed by various superscript letters (A–E) show significant differences among storage time.

Throughout the storage period, the banana slices showed increasing browning values and a decreasing whiteness index (Figure 3). When compared to the treated samples, the whiteness index of raw banana slices decreased significantly over the storage period (*p* ≤ .05) (Figure 3a). The whiteness index among the samples followed the order: Raw < Normal water < Lemon juice < Coconut water. Coconut water‐immersed banana slices contained a significantly higher whiteness index among all treated samples throughout the storage period because coconut water contained ascorbic acid that had reducing properties (Mahayothee et al., 2016). However, Yildiz (2018) found less browning in banana slices treated with ascorbic acid, which agreed with this study. This study was similar to Supapvanich et al. (2020), who found a greater whiteness index in apple wedges submerged in coconut water. Again, the browning value was higher in the raw samples than in the treated samples. The browning value of banana slices followed the order: Coconut water < Lemon juice < Normal water < Raw during the storage periods of 12 days (Figure 3b). There were no significant differences between lemon juice and coconut water‐immersed samples on the 9th and 12th days. These results indicated that the browning value of banana slices during storage was decreased by immersion of lemon juice and coconut water. Lemon juice contains citric acid and ascorbic acid, which have anti‐browning qualities (Moon et al., 2020), whereas coconut water also contains ascorbic acid and salicylic acid, which have the impact of inhibiting browning (Mahayothee et al., 2016). The findings of this study were consistent with the research of Qi et al. (2011), and they found a synergistic impact of edible coatings and anti‐browning chemicals in maintaining lower browning value, better cut‐surface color, and quality of apple wedges. Guerreiro et al. (2017) found that the edible coatings significantly decreased browning in fresh‐cut apples when combined with anti‐browning chemicals (ascorbic acid, citric acid, and sodium chlorite).

### 3.4 Firmness and total soluble solid

**FIGURE 4:**
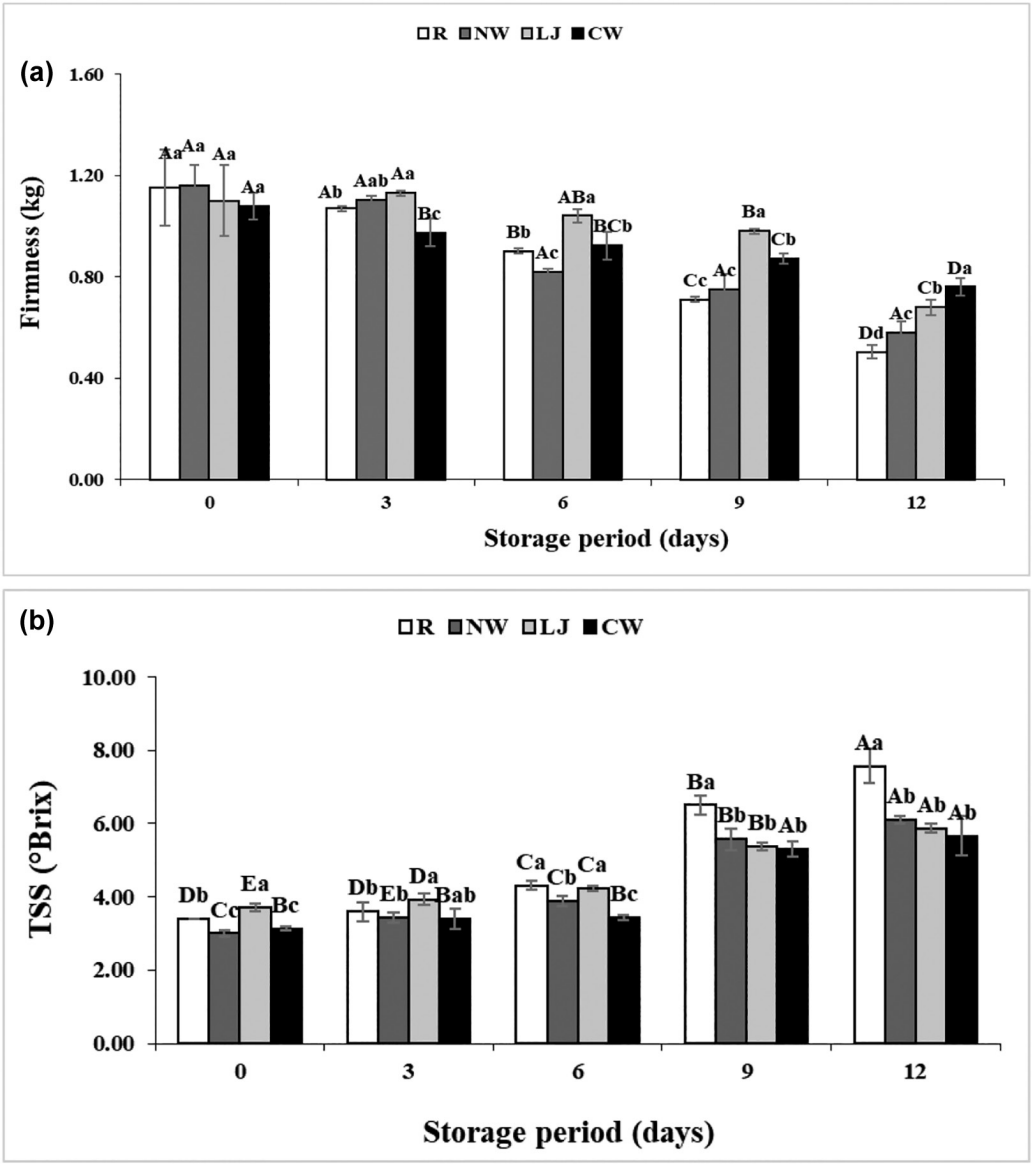
Firmness (a) and Total Soluble Solids (b) of banana slices treated with normal water (NW), lemon juice (LJ), and coconut water (CW) with the raw sample during storage at 4 ± 1°C for 12 days. Means followed by different superscript letters (a–d) vary greatly depending on treatments. Means followed by different superscript letters (A–D) vary greatly depending on storage time.

Firmness, which is correlated with water content, pectin degradation, and metabolic alterations, is a crucial quality criterion for fresh fruit and vegetable consumer appeal. Pectin is one of the major components present in bananas (Duan et al., 2008) and pectin‐degrading enzymes generated during cutting and moisture loss can cause softening or loss of firmness, which is an undesired phenomenon (Qi et al., 2011). Figure 4a reveals that firmness gradually decreased during the storage period. There were no significant differences in the firmness of normal water‐immersed samples during storage periods. This could be attributed to a persistent loss of moisture during the storage period. The firmness was higher in lemon juice on the 3rd, 6th, and 9th days then reduced on the 12th day. The increased moisture loss and breakdown of pectin in banana slices immersed in lemon juice may explain this phenomenon occurring after the 9th day of preservation. However, coconut water‐immersed samples achieved the highest firmness (0.76 kg), and the lowest was found in raw samples (0.50 kg) on the 12th day. Coconut water is a rich source of calcium which could be reacted with pectic acid present in the cell wall of banana slices to form calcium pectate that imparts strength to the cell wall (Olivas et al., 2007). Conversely, without being treated with coconut water, the raw samples had a decrease in firmness with time, which could be attributed to the hydrolysis of pectic acid (Kumar et al., 2018). These results were in agreement with Bico et al. (2009), who revealed the highest rates of firmness loss at control and the lowest rates of firmness loss in treated fresh‐cut banana samples. In addition, the control sample of fresh‐cut bananas had a roughly 47% reduction in firmness, while the dipped and coated samples demonstrated a 15%–20% decrease in firmness (Bico et al., 2010). Furthermore, Alam et al. (2020) observed that edible‐coated dried green banana slices retained firmness better than control samples.

Typical changes in total soluble solids of treated and raw banana slices are shown in Figure 4b. The TSS exhibited a substantial increase in the raw samples compared to the treated samples during the storage period. The TSS content of banana slices at 12th day was in the following order: Coconut water < Lemon juice < Normal water < Raw. There was no significant (*p* ≤ .05) difference in TSS content among all treated samples on the 12th day of storage. Due to reduced respiration and, consequently, sugar metabolism, immersion in natural preservatives may have resulted in a less noticeable increase in TSS content compared to raw produce (Alam et al., 2020). The continual increase in fruit‐free sugars throughout storage was the principal source of the rise in TSS in raw fruits. These results were stable with those of Bico et al. (2009) who found lower TSS in banana slices that were chemically dipped and controlled atmosphere packaged than control samples. Moreover, Alam et al. (2020) also found higher TSS in control bananas than in treated bananas with starch and chitosan solution.

### 3.5 Moisture content and weight loss

**FIGURE 5:**
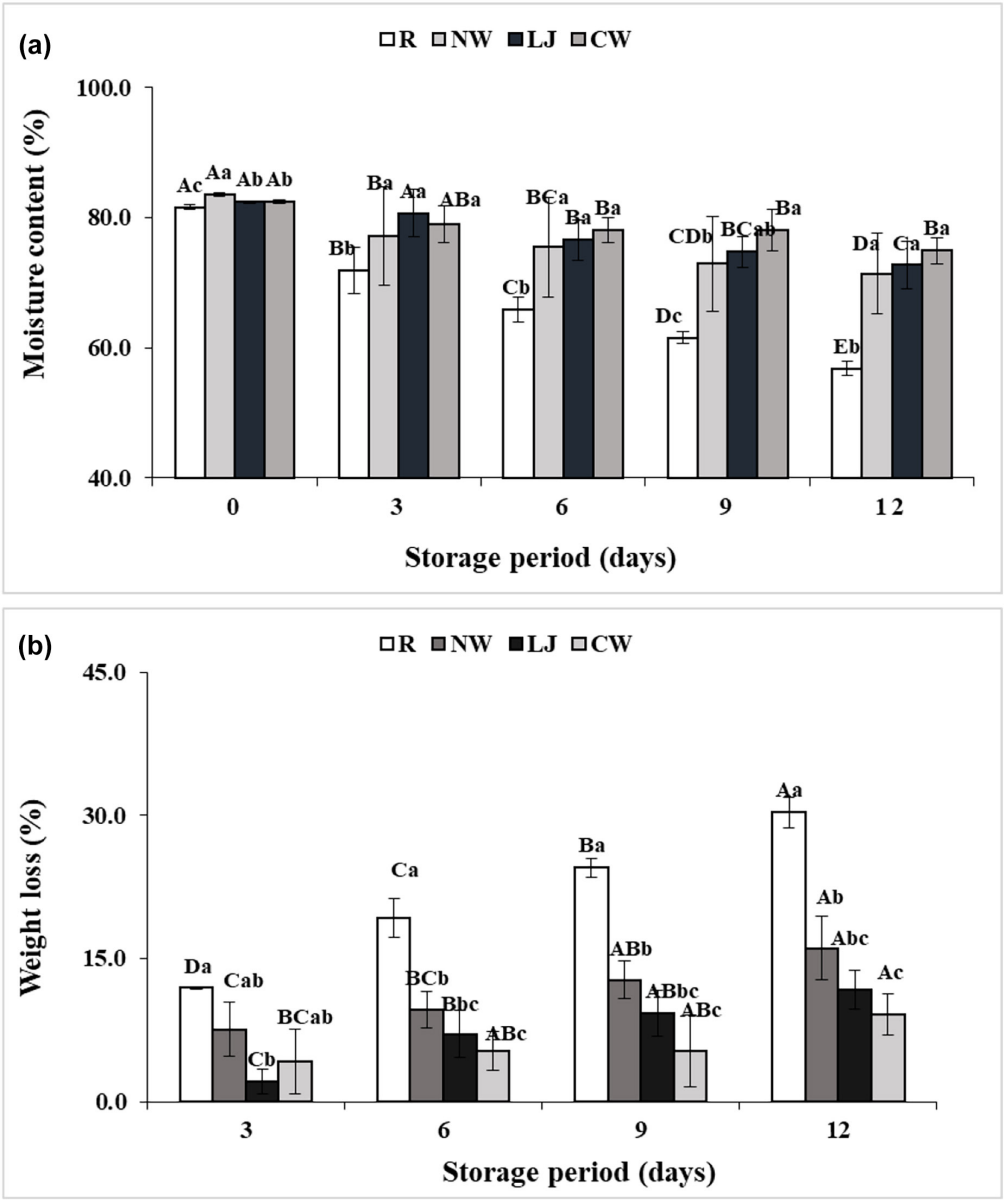
Moisture content (a) and weight loss (b) of banana slices treated with normal water (NW), lemon juice (LJ), and coconut water (CW) with the raw sample during storage at 4 ± 1°C for 12 days. Means with distinct superscript letters (a–c) vary significantly with treatments. Means with distinct superscript letters (A–E) vary significantly with storage time.

Water loss and moisture content are two key elements that influence the fresh‐cut fruit' freshness. It is a measure of freshness and often rises when produce with little processing is stored (Antunes et al., 2010). The moisture content and weight loss of raw and treated banana slices are demonstrated in Figure 5a,b. The samples immersed in coconut water had the maximum moisture content, while the raw samples had the lowest (Figure 5a). According to Mahayothee et al. (2016), coconut water contains a significant number of electrolytes, sugars, and other compounds that can potentially assist in maintaining a high level of moisture in treated banana slices. In Figure 5b, the lowest weight loss of banana slices was observed with the treated samples (Normal water = 16.09%, Lemon juice = 11.76%, and Coconut water = 9.14%), and the raw sample presented the highest loss of weight (30.34%), after 12 days of storage. Due to their barrier characteristics, coconut water, lemon juice, and normal water may prevent browning. According to Moon et al. (2020), lemon juice included citric acid and ascorbic acid, while Mahayothee et al. (2016) reported that coconut water also contained ascorbic acid and salicylic acid. These compounds may aid in forming a protective layer on the food's surface and minimizing moisture loss. Among all treated samples, coconut water‐immersed samples showed the lowest weight loss (9.14%) on the 12th day of storage. This result was compatible with research by Guerreiro et al. (2017) and Liu et al. (2016), who found that the least amount of weight loss of coated fresh‐cut apples occurred when anti‐browning chemicals were added to edible coatings. Moreover, these findings were in line with those of Bico et al. (2009), who discovered that chemically dipped and packaged banana slices in a controlled environment weighed higher than control samples.

### 3.6 Total phenolic content and total flavonoid content

It is generally accepted that exposure to oxygen, PPO activity, and phenolic compounds are the three primary variables that initiate an enzymatic browning response (Wessels et al., 2014). Table 1 shows the effects of normal water, lemon juice, and coconut water on the total phenolic and total flavonoid content of green banana slices during the storage period (4°C ± 1°C for 12 days). It was found that the total phenolic and flavonoid content were significantly higher in coconut water‐immersed samples than in other treated and raw samples since the initial day of storage and then markedly decreased during the storage period. According to Arivalagan et al. (2018), phenolic chemicals, particularly salicylic acid, and catechin were abundant in coconut water, and also flavonoids like myricetin, kaempferol, and quercetin were most frequently present. Consequently, the elevated concentrations of total phenols and flavonoids observed in banana slices could have originated from coconut water. These polyphenols and flavonoids could stop banana slices from turning brown by stopping enzymes like polyphenol oxidase because of their antioxidant properties. Furthermore, the lemon juice‐immersed sample contained more phenol and flavonoids than normal water‐immersed and raw samples. This was because lemon juice also contained many antioxidant properties (Moon et al., 2020). In agreement with prior investigations, the phenolic content of banana slices packaged in a controlled atmosphere and chemically immersed was found to be higher than that of control samples (Bico et al., 2009). According to González‐Aguilar et al. (2005), preserving the highest possible level of phenol content in freshly cut fruits can help them stay fresh and avoid other unfavorable effects over prolonged cold storage.

**TABLE 1:** Effects of normal water, lemon juice, and coconut water on the total phenol and total flavonoid content of green banana slices during the storage period (4 ± 1°C for 12 days).

Storage period (Days)	Sample							
	TPC (mg GAE/100 g FW)				TFC (mg QE/100 g FW)			
	R	NW	LJ	CW	R	NW	LJ	CW
0	^A^414.00 ± 8.00^b^	^A^360.00 ± 5.29^d^	^A^383.00 ± 4.58^c^	^A^457.67 ± 4.51^a^	^A^69.00 ± 3.61^b^	^A^60.00 ± 2.00^c^	^A^75.00 ± 3.00^a^	^A^80.00 ± 2.00^a^
3	^B^377.00 ± 9.17^b^	^B^335.67 ± 9.07^d^	^B^355.33 ± 4.04^c^	^B^442.00 ± 3.00^a^	^B^53.67 ± 2.08^c^	^AB^57.33 ± 4.73^c^	^B^65.00 ± 3.00^b^	^A^77.67 ± 2.08^a^
6	^C^312.00 ± 5.00^c^	^C^315.33 ± 4.04^c^	^C^337.67 ± 3.79^b^	^C^426.33 ± 4.16^a^	^C^40.00 ± 2.00^d^	^B^52.00 ± 3.61^c^	^C^58.33 ± 2.52^b^	^B^69.33 ± 2.52^a^
9	^D^289.67 ± 7.02^d^	^C^307.00 ± 2.00^c^	^D^326.00 ± 4.36^b^	^D^406.33 ± 4.51^a^	^D^28.67 ± 3.51^d^	^C^34.33 ± 3.06^c^	^D^50.33 ± 1.15^b^	^C^62.00 ± 2.65^a^
12	^E^275.00 ± 3.61^d^	^D^291.00 ± 2.00^c^	^E^312.00 ± 3.61^b^	^E^392.67 ± 4.73^a^	^E^16.33 ± 4.16^d^	^D^25.67 ± 3.06^c^	^E^39.00 ± 2.00^b^	^D^55.67 ± 3.51^a^

*Note*: Values are presented as mean ± SD. Means followed by different superscript letters (a–d) in each raw are significantly different among treatments (*p* ≤ .05). Means followed by different superscript letters (A–E) in each column are significantly different among storage time (*p* ≤ .05).

Abbreviations: CW, coconut water‐immersed banana slices; LJ, lemon juice‐immersed banana slices; NW, normal water‐immersed banana slices; R, raw banana slices.

### 3.7 Antioxidant activity assay

The DPPH scavenging activity was higher in coconut water‐immersed banana slices than in other samples during the storage period (Table 2). This phenomenon could potentially be attributed to the enhanced antioxidant and scavenging capabilities of coconut water (Supapvanich et al., 2020). Additionally, DPPH scavenging activity was higher in lemon juice‐immersed banana slices than in normal water and raw samples during the storage period of up to 12 days (Table 2). This could be attributed to the elevated concentration of phenols and flavonoids in lemon juice. However, it seems that anti‐browning treatment may increase the antioxidant activity of banana slices. This result was in tune with that of Alam et al. (2020), who found significantly higher levels of DPPH inhibition in the case of coated banana slices than in control samples. In another research, Supapvanich et al. (2020) observed that coconut water inhibited the oxidative enzymatic browning of apple wedges during the cold storage period (up to 9 days). In addition, Oms‐Oliu et al. (2008) demonstrated the use of an edible coating to increase antioxidant activity in fruits, such as pears.

**TABLE 2:** Effects of normal water, lemon juice, and coconut water on the DPPH scavenging activity (%) of green banana slices during the storage period (4 ± 1°C for 12 days).

Storage period (Days)	Sample			
	R	NW	LJ	CW
0	^A^45.39 ± 3.10^c^	^A^48.73 ± 2.03^c^	^A^57.06 ± 1.11^b^	^A^66.06 ± 1.08^a^
3	^B^36.88 ± 1.57^d^	^B^43.68 ± 2.74^c^	^B^53.43 ± 2.07^b^	^A^62.34 ± 1.28^a^
6	^C^25.11 ± 1.81^d^	^C^36.24 ± 1.03^c^	^C^48.92 ± 1.67^b^	^B^56.92 ± 1.12^a^
9	^D^15.45 ± 0.84^d^	^D^25.57 ± 0.45^c^	^D^45.78 ± 1.03^b^	^C^49.20 ± 3.22^a^
12	^D^12.01 ± 2.09^d^	^E^17.89 ± 0.64^c^	^E^32.10 ± 1.94^b^	^D^38.68 ± 3.26^a^

*Note*: Values are presented as mean ± SD. Means followed by different superscript letters (a–d) in each row are significantly different among treatments. Means followed by different superscript letters (A–E) in each column are significantly different among storage time.

Abbreviations: CW, coconut water‐immersed banana slices; LJ, lemon juice‐immersed banana slices; NW, normal water‐immersed banana slices; R, raw banana slices.

### 3.8 Polyphenol oxidase activity

**FIGURE 6:**
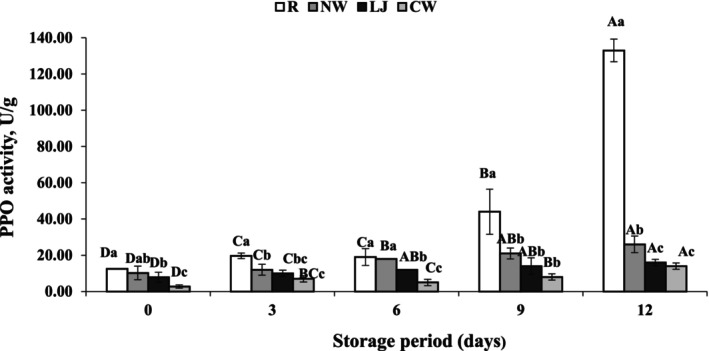
Polyphenol oxidase activity of banana slices treated with normal water (NW), lemon juice (LJ), and coconut water (CW) with the raw sample during storage at 4°C ± 1°C for 12 days. Means followed by various superscript letters (a–c) show significant differences among treatments. Means followed by various superscript letters (A–D) show significant differences among storage time.

Browning of numerous fruits and vegetables, including bananas, is thought to be primarily caused by polyphenol oxidase oxidizing phenolic substrates (Nguyen et al., 2003). In this study, the PPO activity was, therefore, investigated in treated and untreated banana slices during a refrigerated storage period of up to 12 days (Figure 6). The PPO activities of banana slices immersed in lemon juice, coconut water, and normal water were increased slightly during storage periods. The PPO activity of coconut water‐immersed and lemon juice‐immersed banana slices ranged from 2.73 to 14.00 U/g and 7.80 to 16.00 U/g up to 12 days, respectively. The PPO activity increased from 10.20 to 26.00 U/g during the storage of normal water‐immersed samples. Whereas the activity drastically increased from 12.50 to 133.00 U/g in the raw samples. The inhibition power of lemon juice and coconut water was higher than those of others due to the presence of ascorbic acid and phenolic compounds, respectively (Arivalagan et al., 2018; Moon et al., 2020). Furthermore, the increased amount of PPO activity in raw samples might be attributable to the sample's unprotected state toward enzymatic reaction (Bico et al., 2009). This outcome was comparable to that of Yildiz (2018), who found that banana samples treated with ascorbic acid had the lowest PPO activity when compared to the other treatments—a sign of decreased browning. Supapvanich et al. (2020) also observed the lowest PPO activity of coconut water‐immersed apple wedges than control samples.

### 3.9 Correlation of L* value, whiteness index, browning value, TPC, TFC, DPPH scavenging activities, and PPO activity

Pearson's correlation coefficient (*r*) was analyzed to determine the relation between the L* value, whiteness index, browning value, TPC, TFC, DPPH scavenging activities, and PPO activity of banana slices (Table 3). The results showed that the L* value had a higher positive correlation with the whiteness index (*r* = .993) and a negative correlation with the browning value (*r* = −.888). The browning value, on the other hand, had a negative correlation (*r* = −.919) with the whiteness index and a positive correlation (*r* = .860) with PPO activity. The TPC, TFC, and DPPH scavenging activity exhibited a high positive correlation (*r* ≤ .847); however, their correlation with the PPO activity was substantially negative (*r* ≥ −.549). They also have a negative correlation with the browning value and a positive correlation with the L* value and whiteness index. These findings were in line with those of Kaewjumpol et al. (2021), who discovered that in banana blossoms, there was a positive association (*r* = .954) between browning score and PPO activity and a negative correlation (*r* = −.957) between L* value and PPO activity. Furthermore, it was discovered that there was a negative correlation with the browning value and a positive correlation with TPC, DPPH scavenging activity, and L* value (Kaewjumpol et al., 2021).

**TABLE 3:** Pearson's correlation coefficient (*r*) among banana slices L* value, whiteness index, browning value, TPC, TFC, DPPH scavenging activities, and PPO activity.

Variables	L*	WI	BV	TPC	TFC	DPPH	PPO activity
L*	1.000						
WI	0.993	1.000					
BV	−0.888	−0.919	1.000				
TPC	0.716	0.707	−0.619	1.000			
TFC	0.897	0.889	−0.797	0.884	1.000		
DPPH	0.867	0.862	−0.766	0.847	0.958	1.000	
PPO activity	−0.738	−0.738	0.860	−0.549	−0.686	−0.664	1.000

Abbreviations: BV, browning value; DPPH, DPPH scavenging activity; PPO activity, polyphenol oxidase activity; TFC, total flavonoid content; TPC, total phenolic content; WI, whiteness index.

### 3.10 Microbial analysis

Table 4 indicates the effect of natural anti‐browning agents on the microbial growth of fresh‐cut banana slices on different days of refrigerated storage. On the initial day, the maximum microbial load was found in raw banana slices (2.43 × 10^3^ CFU/mL) compared to normal water‐immersed (1.28 × 10^3^ CFU/mL), lemon juice‐immersed (0.99 × 10^3^ CFU/mL), and coconut water‐immersed (1.11 × 10^3^ CFU/mL) banana slices. On the 12th day of storage, the total plate count of raw and normal water‐immersed banana slices was 7.00 × 10^7^ CFU/mL and 5.53 × 10^6^ CFU/mL, respectively. On the same day, the total plate count of lemon juice‐immersed and coconut water‐immersed samples was 3.30 × 10^4^ CFU/mL and 3.5 × 10^4^ CFU/mL, respectively. It proved that microbial load was higher in raw samples than in treated samples. Besides that, the treated samples showed slower bacterial growth when they were refrigerated compared to the fresh samples. Additionally, the microbial load was significantly increased in all samples during storage. According to Jacxsens et al. (2002), the critical limit for total plate count for vegetables was 10^8^ CFU/g and our results of all samples were below the critical limit during the storage of up to 12 days. However, the total plate count of raw samples was near the critical limit because it was a better medium for the growth of microorganisms. The microbial load count of normal water‐immersed samples was under a limit during the storage days because rinsing with normal water can lower microbial growth. However, lemon juice and coconut water contain various types of acids like citric acid, ascorbic acid, and salicylic acid (Arivalagan et al., 2018; Moon et al., 2020). This acid was said to give coats antimicrobial properties and help to keep microbes from growing on fresh‐cut apples (Kumar et al., 2018). Bico et al. (2009) showed that chemical dip + controlled atmosphere packaging + coated banana samples inhibited microbial growth than control one. Furthermore, according to the study by Doyle (2009), the lower temperature may slow down microbial growth, which would reduce the respiration rate and extend the shelf‐life of fresh‐cut items.

**TABLE 4:** Microbial load (total plate count × colony‐forming unit per milliliter [CFU/mL]) of treated and untreated banana slices during refrigerated storage.

Storage period (Days)	Sample (CFU/mL)			
	R	NW	LJ	CW
0	^C^2.43 × 10^3a^	^C^1.28 × 10^3b^	^B^0.99 × 10^3b^	^B^1.11 × 10^3b^
6	^B^4.21 × 10^5a^	^B^2.55 × 10^4b^	^B^1.03 × 10^3c^	^B^1.27 × 10^3d^
12	^A^7.00 × 10^7a^	^A^5.53 × 10^6b^	^A^3.30 × 10^3c^	^A^3.50 × 10^3c^

*Note*: Means followed by various superscript letters (a–d) show significant differences among treatments. Means followed by various superscript letters (A–C) show significant differences among storage time.

Abbreviations: CW, coconut water‐immersed banana slices; LJ, lemon juice‐immersed banana slices; NW, normal water‐immersed banana slices; R, raw banana slices.

## 4 CONCLUSION

This study aimed to identify the most effective natural anti‐browning agent for preserving fresh‐cut green bananas during 12‐day refrigerated storage at 4°C. Treated samples showed superior quality compared to untreated ones. Coconut water immersion notably maintained visual appeal, firmness, and low total soluble solid levels throughout storage, with minimal weight loss (9.14%). Coconut water‐treated samples exhibited consistently higher phenol, flavonoid, and antioxidant levels, and effectively deactivated polyphenol oxidase activity. Microbial growth was significantly reduced in coconut water‐treated samples. Overall, immersing fresh‐cut bananas in coconut water extended their shelf‐life and prevented browning effectively. This suggests coconut water as a promising natural solution for preserving freshness in fresh‐cut fruits and vegetables, aiding industry efforts to enhance the shelf‐life and quality.

## AUTHOR CONTRIBUTIONS

**Shampa Sarkar:** Investigation (equal); methodology (equal); writing – original draft (equal). **Sumaia Akhter:** Data curation (equal); writing – review and editing (equal). **Joysree Roy:** Conceptualization (equal); supervision (equal); writing – review and editing (equal). **Md. Abdul Wazed:** Writing – review and editing (equal). **Raihan Abedin:** Methodology (equal). **Suvrow Neogie:** Methodology (equal). **Khairul Bashar Mishat:** Methodology (equal). **Md. Sazzat Hossain Sarker:** Supervision (equal).

## CONFLICT OF INTEREST STATEMENT

The authors affirm that there is no conflict of interest among them.

## Data Availability

Data will be provided by the corresponding author upon request.
